# Respectful maternity care among midwifery graduates trained under competency-based versus conventional curricula in Ethiopia: a comparative cross-sectional study

**DOI:** 10.1186/s12909-026-10661-5

**Published:** 2026-10-24

**Authors:** Awoke Giletew Wondie, Matthias Siebeck, Tegbar Yigzaw Sendekie, Martin R. Fischer, Markus Berndt

**Affiliations:** 1 CIH LMU, Center for International Health, LMU University Hospital, LMU Munich, Leopoldstraße 5, München, 80802 Germany; 2 Educational Development and Quality Centre (EDQC), University of Global Health Equity (UGHE), Kigali, Rwanda; 3 Institute of Medical Education, LMU University Hospital, LMU Munich, München, Germany; 4 Health Workforce Initiatives, Mastercard Foundation, Kigali, Rwanda

**Keywords:** Clinical competence, Curriculum, Ethiopia, Midwifery

## Abstract

**Background:**

In Ethiopia, midwifery education emphasizes theory over practice, limiting clinical competence, including professional behaviors. Debre Tabor University (DTU) adopted a competency-based curriculum in 2013 to address this. This study examined whether graduates trained under competency-based and conventional curricula differed in their observed provision of respectful maternity care (RMC).

**Methods:**

A comparative cross-sectional study assessed observed mistreatment during care provided by 68 BSc midwifery graduates (32 trained under a competency-based curriculum and 36 under a conventional curriculum) from third-generation Ethiopian universities. Care was assessed through direct observation using a validated tool. Mean mistreatment scores were compared using t-tests, and the between-group difference was visualised using a Gardner–Altman plot.

**Results:**

Overall, the mean mistreatment score was 34.6%. Conventional curriculum graduates had a higher mean score than competency-based curriculum graduates (38.1% versus 30.8%). The difference was statistically significant, t(66) = − 3.75, *p* < .001, with an absolute mean difference of 7.3% points and Cohen’s d = − 0.91 for competency-based versus conventional graduates. Descriptively, poor-rapport scores (36.7% versus 46.5%) and non-response to medical requests (15.6% versus 30.5%) were lower in the competency-based group. Both groups had recorded deficiencies in consent, supportive care, and privacy, as well as instances of verbal abuse.

**Conclusion:**

Competency-based curriculum background was associated with lower observed mean mistreatment scores during labour and childbirth. This association does not establish a causal curriculum effect, and lower mistreatment scores do not demonstrate comprehensive RMC. Deficiencies observed in both groups warrant further attention. Longitudinal and multilevel research is needed to examine the contributions of preservice education and workplace conditions.

**Supplementary Information:**

The online version contains supplementary material available at https://doi.org/10.1186/s12909-026-10661-5.

## Background

Childbirth experiences significantly impact women’s health decisions and emotional well-being. While RMC is a fundamental human right, global studies report 15–98% of women experience mistreatment during facility births, ranging from physical abuse to non-consented care [1]. The World Health Organization (WHO) defines RMC as “care that is organized and delivered to all women in a way that upholds their dignity, privacy, and confidentiality, protects them from harm and mistreatment, and promotes informed choice and continuous support during labor and childbirth” [2].

Global efforts and WHO guidelines have elevated RMC as a priority in maternal health [3]. Recent research highlights RMC’s dual importance: preventing mistreatment while promoting positive practices like skilled maternal care. Quality RMC improves health outcomes, increases service utilization, and reduces maternal depression. However, measurement challenges persist due to inconsistent definitions and assessment tools [4].

In Ethiopia, university midwifery education’s theoretical focus limited graduates’ practical competencies. In 2013, DTU adopted a competency-based integrated curriculum incorporating problem-based learning, early clinical exposure, simulation-based education, and community-based education [7, 8, 9]. While evidence supports the role of active, community-oriented education in shaping students’ empathy, diagnostic skills, and communication, few studies have linked curriculum design in preservice education (PSE) to intermediate health outcomes [8, 9].

Evidence of improved clinical competence following educational reform does not establish whether graduates demonstrate respectful professional behaviours during workplace care. An important question is whether differences in preservice curriculum are associated with observed practices concerning consent, privacy, supportive care, responsiveness, and freedom from abuse and discrimination. These practices represent intermediate educational outcomes expressed through care delivery, rather than direct measures of maternal or newborn health outcomes. Their assessment helps examine the relationship between preservice education and clinical practice quality while recognising the potential influence of workplace conditions. This study therefore compared selected observable dimensions of respectful maternity care, assessed through mistreatment indicators, among recently deployed midwives trained under competency-based and conventional curricula in Ethiopia.

### Objectives

This study compared the observed respectful maternity care performance of recently deployed midwives trained under competency-based and conventional preservice curricula in Ethiopia and examined whether curriculum background was associated with differences in professional behaviour during labour and childbirth.

## Methods

### Study design

A comparative cross-sectional study was conducted to compare respectful maternity care performance between graduates trained under competency-based and conventional preservice curricula. Curriculum exposure was determined by the university and curriculum under which each midwife had been trained, whereas performance was assessed in the public health facility where the graduate was employed.

This analysis draws on the same cohort of 68 midwifery graduates, participating women, health facilities, and data-collection period as a previously published analysis of quality labour and delivery care [10]. The present study examines a separate outcome, respectful maternity care and mistreatment during childbirth, assessed using a distinct validated observation instrument [5].

### Setting

The study was conducted in Ethiopia among recently deployed Bachelor of Science midwifery graduates from third-generation public universities. Debre Tabor University introduced a competency-based, integrated midwifery curriculum in 2013, whereas the comparison universities continued to implement the conventional curriculum for the graduating cohort included in this study.

Third-generation universities were purposively selected as the comparison context because they were established during the same period as Debre Tabor University and operate within the same national public higher-education governance and resource-allocation framework. Universities established within the same generation are broadly comparable in terms of staffing structures, educational facilities and resources, clinical practice sites, institutional budgets, and student support services. They also deliver the same Bachelor of Science midwifery qualification under the national higher-education and professional regulatory system.

This selection strategy was intended to minimise contextual differences between institutions so that the main educational distinction between the groups was the type of preservice curriculum implemented during the participants’ training. Data were collected from August 26 to October 12, 2024.

### Participants

The study included recently deployed Bachelor of Science midwifery graduates who had passed the national licensure examination, obtained employment within one year of graduation, had no more than one year of professional experience, and were assigned to provide labour and childbirth care. Graduates who had received relevant in-service training, including training in respectful maternity care, were excluded.

These criteria were applied to graduates from both the competency-based and conventional-curriculum groups. Restricting the study to recently deployed graduates from the same generation of public universities reduced variation related to institutional context, professional experience, post-graduation training, degree requirements, and national licensure standards.

The study focused on eligible midwives, not the women observed as the women were not interviewed or surveyed; therefore, the findings represent observer-recorded care practices rather than women’s self-reported experiences. Cases involving other providers or procedures beyond midwives’ scope (e.g., cesarean deliveries) were excluded.

### Variables and operational definitions

The primary outcome was the overall observed mistreatment score. Indicators were organised into four reporting domains corresponding to selected categories of the mistreatment typology [4].

Abuse and discrimination comprised physical abuse, verbal abuse, and stigma or discrimination. These categories included the use of force or physical restraint, harsh language, threats or blaming, and discriminatory treatment related to women’s characteristics. The indicator “at least one observed form of abuse or discrimination” represented the occurrence of any of these categories during an observed encounter.

Failure to meet professional standards of care comprised episiotomy without informed consent, vaginal examinations without information or permission, non-provision of requested pain relief, absence of the midwife at birth, and failure to respond to requests for medical attention.

Poor rapport comprised omissions in supportive care and attention to women’s preferences. The measured indicators were failure to offer a labour companion, lack of easy access to water or oral fluids, failure to inform women that they could mobilise combined with no observed mobilisation, and failure to ask about the preferred birthing position.

Health-system conditions comprised bedsharing, absence of privacy measures, instructions to women to clean bodily fluids, and requests for bribes, informal payments, or gifts. These indicators concerned care conditions and facility practices and were not interpreted solely as individual provider behaviours.

Care was recorded independently by two trained observers from the initial assessment through childbirth and up to two hours postpartum. Observer scores were averaged for analysis. Individual indicators were reported using their applicable denominators: women for woman-level indicators, episiotomies for episiotomy-consent indicators, vaginal examinations for examination-related indicators, and women requesting pain relief for non-provision of requested pain relief.

As described in the scoring procedure, the overall score represented the proportion of applicable mistreatment indicators recorded, ranging from 0 to 1, with higher values indicating greater observed mistreatment. Scores were also expressed as percentages. The mean mistreatment score therefore describes the average proportion of applicable indicators recorded, not the proportion of women experiencing any mistreatment.

### Data sources/ measurement

Respectful maternity care was the overarching construct informing this study. Consistent with the WHO definition, it encompasses dignity, privacy, confidentiality, protection from harm and mistreatment, informed choice, and continuous support [2]. Selected dimensions were assessed through direct observation using the validated labour-observation instrument [5]. Findings were organised using relevant categories of the mistreatment typology developed by Bohren and colleagues [4].

The tool included three components: (1) admission form; (2) incident report form; and (3) childbirth, interventions and discharge form. The admission form captures screening questions, and sociodemographic information about the woman. Incident report form was completed if, and only if, one of the following events occurred: physical abuse, verbal abuse, stigma and discrimination, or a vaginal examination. If one of these incidents occurred, then the form was completed and submitted immediately. This form could have been completed and submitted multiple times, in case of multiple instances of physical abuse, verbal abuse, stigma and discrimination, or vaginal examination, or never, in case none of these instances occurred. Childbirth, interventions and discharge form was completed at the end of the period of observation (one per woman). This form captured information about what events transpired throughout the woman’s labour and childbirth period, including pain relief, mobilization, fluids, labour companionship, demands from healthcare providers, fees, neglect, the childbirth outcome, status of the baby, privacy during childbirth, availability of beds, maternal interventions and informed consent, newborn interventions, referral/discharge, and outcome of observation [5].

Data collectors were master ‘s-level clinical midwives with over one year of maternal/newborn care experience and no affiliation with study sites. A five-day training in Gondar (August 19–23, 2024) covered study protocols, tools, mobile data collection (Kobo Collect), and ethical principles. The training involved presentations, role-play, and practice sessions to enhance interrater reliability.

#### Data collection field work

Study procedures involved screening eligible midwives and inviting labouring women in established labour to participate. Observations began with initial evaluation and continued through childbirth and up to two hours postpartum. Two trained observers independently and unobtrusively documented care, using average scores for analysis. Only cases where the primary provider was the selected midwife were included. Data were collected using the Kobo Collect mobile platform.

#### Reliability and internal consistency

Interrater reliability, assessed via intraclass correlation coefficient (ICC), was excellent (ICC = 0.84; 95% CI: 0.74–0.90; *p* < .001), with Cronbach’s alpha of 0.64, indicating modest reliability for this study.

### Sample size and sampling

The sample size was calculated for a two-sided independent-samples comparison, assuming 80% power, (α = 0.05), equal group allocation, and a standardized mean difference of approximately Cohen’s (d = 0.71). This calculation resulted in a minimum requirement of 32 participants per group.

Of the 37 recent Debre Tabor University graduates initially considered, 32 met the eligibility criteria, and all eligible graduates were included. Five were excluded because they were unemployed, had changed careers, or were working outside the study area. To allow for possible non-participation, 36 eligible conventional-curriculum graduates were randomly selected from the comparison-university graduate registers.

### Statistical methods

Data was analyzed using IBM SPSS v27. Individual mistreatment indicators were summarised using frequencies and percentages based on their applicable denominators. Mean domain mistreatment scores and the overall mean mistreatment score were calculated and presented on a 0–1 scale and as percentages. Higher scores represented a greater occurrence of observed mistreatment-related behaviours or omissions. Independent *t* tests compared graduates from competency-based and conventional curricula, with effect sizes (Cohen’s *d*) visualized by Gardner–Altman plots.

Multiple comparison corrections were applied using the Bonferroni, Holm–Bonferroni, and false discovery rate methods to account for the analysis of four related primary outcomes. The primary outcome remained statistically significant under all adjustments.

## Results

### Participants

#### Service providers

A total of 68 midwifery service providers were observed. Most (97.1%) had 6–12 months of experience, aligning with the time since earning their BSc (Table 1).

**Table 1:** Characteristics of midwives observed by type of curriculum in preservice education (*N = 68*)

Variables		CCGs (*n* = 36)		CBCGs (*n* = 32)		Total (*N* = 68)	
		N	%	n	%	n	%
Age Mean ± *SD* = 24.58 ± 0.814	< 25	14	38.9	14	43.7	28	41.2
	25–29	22	61.1	18	56.3	40	58.8
Sex	Male	16	44.4	21	65.6	37	54.4
	Female	20	55.6	11	34.45	31	45.6
Marital status	Married	9	25.0	6	18.7	15	22.1
	Not married	27	75.0	26	81.3	53	77.9
Experience	0–5 months	2	5.5	0	0.0	2	2.9
	6–12 months	34	94.2	32	100	66	97.1
Workplace by region/City administrations	Addis Ababa	11	30.5	12	37.5	23	33.8
	Amhara	14	38.9	9	28.1	23	33.8
	Dire Dawa	4	11.2	3	9.4	7	10.3
	Somali	2	5.5	2	6.2	4	5.9
	Central Ethiopia	4	11.1	4	12.5	8	11.7
	Southern Ethiopia	1	2.7	2	6.2	3	4.4
Type of facility	Referral hospital	5	13.9	4	12.5	9	13.2
	General hospital	6	16.7	4	12.5	10	14.7
	Primary hospital	7	19.4	5	15.6	12	17.7
	Health center	18	50.0	19	59.4	37	54.4

*CCGs* Conventional curriculum graduates, *CBCGs* Competency-based curriculum graduates

#### Client and facility characteristics

Sixty-eight laboring women were included, most aged 25–34 years (86.7%) and from urban areas (64.7%). All observations occurred in public facilities, primarily health centers (65.6%), with comparable representation from both groups.

### Main results

At least one observed form of abuse or discrimination occurred among 35 of the 68 women (51.4%). This proportion was higher among women cared for by conventional curriculum graduates than among those cared for by competency-based curriculum graduates (55.5% versus 46.8%). This indicator represents the abuse and discrimination domain and should not be interpreted as the overall mistreatment score (Table 2).

**Table 2:** Observed mistreatment of women during labor and delivery care by a curriculum type in PSE, Ethiopia (*N* = 68)

Actions	CCG (*n* = 36)		CBCG (*n* = 32)		Total (*N* = 68)	
	*n*	%	*n*	%	*n*	%
At least one observed form of abuse or discrimination (%)	20	55.5	15	46.8	35	51.4
Any physical abuse	8	22.2	6	18.7	14	20.6
Any verbal abuse	16	44.4	13	40.6	29	42.6
Any stigma or discrimination	2	5.5	2	6.3	4	5.9
Failure to meet professional standards of care (%)	**49.7**		**41.9**		**45.5**	
Lack of informed consent and confidentiality						
Episiotomy	7	19.4	5	15.76	12	17.6
Not consented	7	100.0	5	100.0	12	100.0
All vaginal examinations	88	-	84	-	172	-
Not informed or no permission	64	72.7	55	65.5	119	69.2
Refusal to provide pain relief						
Woman requested pain relief	11	30.5	7	21.8	18	29.4
Did not receive pain relief ^b^	5	45.6	2	28.6	7	38.9
Neglect and abandonment						
Midwives absent when the baby came out	0	0.0	0	0.0	0	0.0
The woman requests medical attention from a midwife that was not responded	11	30.5	5	15.6	16	23.5
Poor rapport between women and midwife (%)	**46.5**		**36.7**		**41.9**	
Lack of supportive care						
Not offered to have a labor companion during labor and birth	21	58.3	14	43.7	35	51.5
No easy access to water or oral fluids	4	11.1	2	6.2	6	8.8
Woman not told she could mobilize during labor, and did not mobilize	9	25.0	5	15.6	14	20.6
Not asked for her preferred birthing position	33	91.6	26	81.2	59	86.7
Health systems and conditions (%)	**17.4**		**14.1 **		**15.8**	
Supply constraint						
At any time, the woman shared a bed with another woman or women	0	0.0	0	0.0	0	0.0
Lack of privacy						
Curtains, partitions, or other measures not used to provide privacy for the woman throughout labor or childbirth or postpartum periods	25	69.4	18	56.2	43	63.2
Facility culture						
Woman instructed to clean up blood, urine, feces, or amniotic fluid	0	0.0	0	0.0	0	0.0
Midwife suggested or asked the woman or companion for a bribe, informal payment, or gift	0	0.0	0	0.0	0	0.0
Overall mean mistreatment score, % (95% CI)	**38.09** **(35.4–40.8)**		**30.80** **(27.9–33.7)**		**34.66** **(32.5–36.8)**	

*CBCGs* Competency-based Curriculum Graduates, *CCG* Conventional Curriculum Graduates

ªthe denominator is across all examinations, ^b^the denominator is among those requested

#### Professional standards

Among the 12 observed episiotomies, none was preceded by documented informed consent. Of the 172 observed vaginal examinations, consent was documented for 53 examinations (30.8%), while 119 (69.2%) were conducted without documented consent or permission. Among the 18 women who requested pain relief, 7 (38.9%) did not receive it. The proportion was numerically higher in encounters involving conventional curriculum graduates than in those involving competency-based curriculum graduates (45.6% vs. 28.6%). Similarly, failure to respond to a woman’s request for medical attention was observed in 30.5% of encounters involving conventional curriculum graduates and 15.6% of encounters involving competency-based curriculum graduates (Table 2).

#### Rapport

Although 91.2% of women had easy access to water or oral fluids, 35 of 68 women (51.5%) were not offered the option of having a labour companion. In addition, 59 of 68 women (86.7%) were not asked about their preferred birthing position (Table 2).

#### Health system constraints

Privacy measures were recorded in 36.7% of observations. No instances of bedsharing, requests for bribes, or instructions for women to clean bodily fluids were recorded during the observed encounters (Table 2).

#### Overall mistreatment

The overall mean mistreatment score was 34.6% (95% CI: 32.5%–36.8%). The mean score was higher among conventional curriculum graduates than among competency-based curriculum graduates (38.1% versus 30.8%). Indicator frequencies and domain scores are presented in Table 2; the proportion of women with any abuse or discrimination should not be directly ranked against mean domain scores.

#### Comparison of women’s mistreatment

The mean mistreatment score differed significantly between the two groups, t(66) = − 3.75, *p* < .001. Conventional curriculum graduates had a higher mean score (*M* = 0.381, *SD* = 0.080) than competency-based curriculum graduates (*M* = 0.308, *SD* = 0.080), corresponding to an absolute mean difference of 0.073, or 7.3% points (Table 3).

**Table 3:** Comparing the mean score of women’s mistreatments during labor and delivery by a curriculum type, Ethiopia (*N* = 68)

Outcome	Competency-based curriculum (*n* = 32), mean (SD)	Conventional curriculum (*n* = 36), mean (SD)	Mean difference (95% CI)	*p*-value
Overall mistreatment score	0.308 (0.080)	0.381 (0.080)	−0.073 (− 0.112 to − 0.034)	< 0.001

Scores range from 0 to 1; higher scores indicate greater observed mistreatment. The mean difference is competency-based minus conventional curriculum. Two-sided independent-samples t-test with equal variances assumed: t(66) = − 3.75. The p-value is unadjusted

*SD* standard deviation, *CI* confidence interval

#### Effect size estimation

**Fig. 1:**
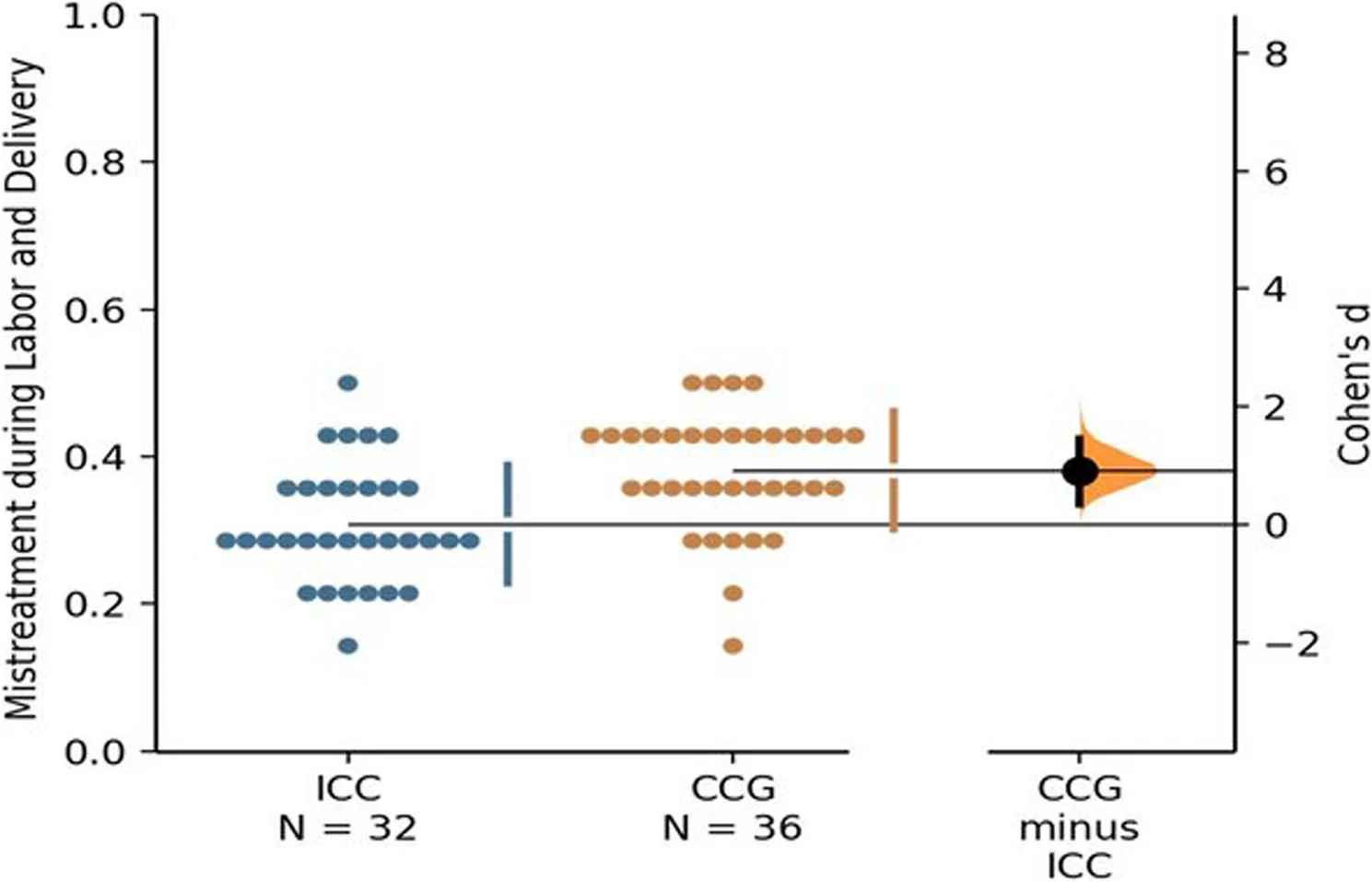
Gardner‒Altman plot visualizing effect size for women’s mistreatment during labor and delivery care by a curriculum type in PSE, Ethiopia (*N* = 68)

The between-group difference in mean mistreatment scores was visualized using a Gardner–Altman plot generated with the DABEST package [11]. Competency-based curriculum graduates had a lower mean score than conventional curriculum graduates (M = 0.308, SD = 0.080, *n* = 32 versus M = 0.381, SD = 0.080, *n* = 36). The mean difference for competency-based versus conventional curriculum graduates was − 0.073 on the 0–1 scale (95% CI: −0.112 to − 0.034), equivalent to a mean score approximately 7.3% points lower. Cohen’s d was − 0.91; the negative direction indicates lower mean mistreatment scores in the competency-based group (Fig. 1).

## Discussion

### Key results

This study compared selected observable dimensions of RMC, assessed through mistreatment indicators, among midwifery graduates working in 29 Ethiopian public health facilities. The overall mean mistreatment score was 34.6%. Conventional curriculum graduates had a higher mean score than competency-based curriculum graduates (38.1% versus 30.8%), with a statistically significant difference of approximately 7.3% points. The reported domain indicators generally favoured the competency-based group descriptively, while deficiencies were recorded in both groups. Statistical significance and the standardised mean difference do not, by themselves, establish patient-level benefit or comprehensive RMC.

### Interpretation

The findings concern selected observable dimensions of respectful maternity care, expressed through mistreatment indicators. Respectful and disrespectful practices may coexist within the same encounter, and absence of recorded mistreatment does not establish comprehensive RMC [1]. Lower mistreatment scores should therefore be interpreted as fewer recorded deficiencies in the assessed dimensions, rather than as a complete measure of respectful care or women’s childbirth experiences.

The observed mean difference of 7.3% points indicates lower mean mistreatment scores among competency-based curriculum graduates in this sample. Experiential learning, simulation, supervised clinical exposure, and feedback are possible educational explanations, but the study did not determine their contribution to the observed association. Workplace culture, supervision, staffing, facility resources, leadership, and organisational norms may also have influenced care. The findings therefore support further investigation of the relationship between preservice education and RMC-related practices; they do not establish effects on patient-reported experience, service utilisation, or maternal and newborn health outcomes.

Recorded deficiencies in both groups, including verbal abuse and inadequate privacy, identify areas for improvement. Their causes cannot be determined from this study. Educational preparation, clinical supervision, and facility conditions warrant consideration as potential targets for further assessment and quality improvement.

The study did not measure communication competence, empathy, ethical reasoning, attitudes, individual curriculum exposure, or fidelity of curriculum implementation as distinct explanatory variables. These factors should therefore be regarded as possible mechanisms rather than demonstrated mediators.

### Comparison with previous studies

Published studies of mistreatment during childbirth differ in their definitions, instruments, and outcome measures [4–6]. The present overall mean mistreatment score of 34.6% represents an average proportion of applicable indicators, not the proportion of women experiencing mistreatment. Direct numerical comparisons with published prevalence estimates are therefore inappropriate. Comparisons should consider both the domains assessed and the way each outcome was calculated.

The recorded omissions of consent, non-provision of requested pain relief, and instances of verbal abuse warrant attention in both curriculum groups. However, this study did not establish whether these deficiencies arose from preservice education, workplace conditions, or their interaction. It cannot determine whether curriculum reform alone or a combination of educational and workplace interventions would improve these practices; those approaches require direct evaluation.

### Limitations

Including graduates from third-generation public universities was intended to improve contextual comparability. Participants had no more than one year of professional experience, had not received relevant in-service training, including RMC training, and had passed the national licensure examination. These restrictions limited eligibility to a defined group but did not demonstrate the absence of selection bias or unmeasured differences between groups.

The use of a validated observation instrument, trained independent observers, and blinding to curriculum background was intended to reduce measurement bias. Averaging the two observers’ scores did not eliminate possible systematic measurement error. The ICC describes observer agreement, while the modest internal consistency (Cronbach’s alpha = 0.64) should also be considered when interpreting the composite score.

Direct observation may have influenced provider behaviour through the Hawthorne effect. Standardised training, observers’ independence from the facilities, confidentiality assurances, unobtrusive observation, and blinding to curriculum background were intended to limit observation-related bias. The magnitude of any change in behaviour, and whether it differed between curriculum groups, were not assessed. The findings should therefore be interpreted as care recorded under observation rather than exact estimates of unobserved routine practice.

The comparative cross-sectional design does not permit causal attribution of the observed differences to curriculum type. Workplace culture, supervision, staffing, facility resources, leadership, organisational norms, regional context, and informal professional socialisation may also have influenced provider behaviour. These factors were not comprehensively measured or adjusted for in the present analysis. Residual confounding may therefore remain, and the findings should be interpreted as an association between curriculum background and observed respectful maternity care performance rather than evidence of a direct curriculum effect.

### Generalizability

The study included graduates working across several public health facilities and geographical settings in Ethiopia. Nevertheless, the modest sample size, the inclusion of competency-based graduates from one university, and the potential influence of unmeasured institutional and workplace factors limit generalisability. The findings may be relevant to comparable settings, but they should not be assumed to apply to all midwifery programmes or other low- and middle-income countries without further investigation.

### Suggestions

Further research could use longitudinal and multilevel designs to examine the contributions of preservice curriculum, institutional characteristics, workplace conditions, and continuing professional development to RMC-related practices. An explanatory sequential mixed-methods design could use qualitative inquiry to explore mechanisms suggested by quantitative findings. Educational and workplace interventions addressing the observed deficiencies warrant evaluation. Future studies should also examine whether differences in observed care practices are associated with patient-reported childbirth experience, trust, subsequent service utilisation, and maternal and newborn outcomes.

## Conclusion

Midwifery graduates trained under the competency-based curriculum had lower observed mean mistreatment scores than those trained under conventional curricula. This association does not establish a causal curriculum effect, and lower scores do not demonstrate comprehensive respectful maternity care. Deficiencies observed in both groups identify areas for improvement. Further research is needed to distinguish the contributions of preservice education, institutional characteristics, and workplace conditions.

## Supplementary Information

Additional file 1: Data set.

Additional file 2: Figure - Study workflow and procedures for delivery observations.

Additional file 3: Table - Background of clients observed and characteristics of health facilities.

## Data Availability

Data is provided within the manuscript or supplementary information files.

## Abbreviations

BSc: Bachelor of Sciences
CBCGs: Competency-based Curriculum Graduates
CCGs: Conventional Curriculum Graduates
DTU: Debre Tabor University
ICC: Intraclass Correlation Coefficient
MCH: Maternal and Child Health
PSE: Preservice Education
WHO: World Health Organization
