# Supplementary material for: Respectful maternity care among midwifery graduates trained under competency-based versus conventional curricula in Ethiopia: a comparative cross-sectional study
Source: BMC Med Educ. 2026 Oct 24;26:1725. doi: 10.1186/s12909-026-10661-5 (PMC13700421; doi:10.1186/s12909-026-10661-5)
Supplement: Supplementary file 1 [file 12909_2026_10661_MOESM2_ESM.docx]

**Supplement 1: Figure 1 Study procedure and workflow for the labor and delivery observations** (adopted by the research team).

**Supervisor**

**Arrives at health facility where service provider works**

**Service provider enrolled in the study**

**Data collectors**

**Completion of screening form**

**Service provider consent**

**Screening & pre-eligibility checks**

**Arrives at health facility where service provider works**

**Observation starts**

**Women arrive at health facility & admitted for childbirth**

**Women in labor**

**Women give birth**

Women

**Women**

**Women screening,**

**Obtain women consent**

**Women enrolled in the study**

**Service provider**

**Labor & Delivery observation form completed,**

**End of data collection: 1 hour postpartum**

**Central data management team**

**Consistency data check**

**Data cleaning**

**Final database**

**Data auditing & monitoring**

**Daily screening and data submission**

**Data consistency report**

**Regular email and phone communication**

Data consistency

Regual communication with data collectors
